# Combined Pharmacotherapy and Cognitive Behavioral Therapy for Adults With Alcohol or Substance Use Disorders: A Systematic Review and Meta-analysis

**DOI:** 10.1001/jamanetworkopen.2020.8279

**Published:** 2020-06-19

**Authors:** Lara A. Ray, Lindsay R. Meredith, Brian D. Kiluk, Justin Walthers, Kathleen M. Carroll, Molly Magill

**Affiliations:** 1 Department of Psychology, University of California, Los Angeles; 2 Yale School of Medicine, New Haven, Connecticut; 3 Center for Alcohol and Addiction Studies, Brown University, Providence, Rhode Island

## Abstract

**Importance:**

Substance use disorders (SUDs) represent a pressing public health concern. Combined behavioral and pharmacological interventions are considered best practices for addiction. Cognitive behavioral therapy (CBT) is a first-line intervention, yet the superiority of CBT compared with other behavioral treatments when combined with pharmacotherapy remains unclear. An understanding of the effects of combined CBT and pharmacotherapy will inform best-practice guidelines for treatment of SUD.

**Objective:**

To conduct a meta-analysis of the published literature on combined CBT and pharmacotherapy for adult alcohol use disorder (AUD) or other SUDs.

**Data Sources:**

PubMed, Cochrane Register, MEDLINE, PsychINFO, and Embase databases from January 1, 1990, through July 31, 2019, were searched. Keywords were specified in 3 categories: treatment type, outcome type, and study design. Collected data were analyzed through September 30, 2019.

**Study Selection:**

Two independent raters reviewed abstracts and full-text articles. English language articles describing randomized clinical trials examining CBT in combination with pharmacotherapy for AUD and SUD were included.

**Data Extraction and Synthesis:**

Inverse-variance weighted, random-effects estimates of effect size were pooled into 3 clinically informative subgroups: (1) CBT plus pharmacotherapy compared with usual care plus pharmacotherapy, (2) CBT plus pharmacotherapy compared with another specific therapy plus pharmacotherapy, and (3) CBT added to usual care and pharmacotherapy compared with usual care and pharmacotherapy alone. Sensitivity analyses included assessment of study quality, pooled effect size heterogeneity, publication bias, and primary substance moderator effects.

**Main Outcomes and Measures:**

Substance use frequency and quantity outcomes after treatment and during follow-up were examined.

**Results:**

The sample included 62 effect sizes from 30 unique randomized clinical trials that examined CBT in combination with some form of pharmacotherapy for AUD and SUD. The primary substances targeted in the clinical trial sample were alcohol (15 [50%]), followed by cocaine (7 [23%]) and opioids (6 [20%]). The mean (SD) age of the patient sample was 39 (6) years, with a mean (SD) of 28% (12%) female participants per study. The following pharmacotherapies were used: naltrexone hydrochloride and/or acamprosate calcium (26 of 62 effect sizes [42%]), methadone hydrochloride or combined buprenorphine hydrochloride and naltrexone (11 of 62 [18%]), disulfiram (5 of 62 [8%]), and another pharmacotherapy or mixture of pharmacotherapies (20 of 62 [32%]). Random-effects pooled estimates showed a benefit associated with combined CBT and pharmacotherapy over usual care (*g* range, 0.18-0.28; *k = *9). However, CBT did not perform better than another specific therapy, and evidence for the addition of CBT as an add-on to combined usual care and pharmacotherapy was mixed. Moderator analysis showed variability in effect direction and magnitude by primary drug target.

**Conclusions and Relevance:**

The present study supports the efficacy of combined CBT and pharmacotherapy compared with usual care and pharmacotherapy. Cognitive behavioral therapy did not perform better than another evidence-based modality (eg, motivational enhancement therapy, contingency management) in this context or as an add-on to combined usual care and pharmacotherapy. These findings suggest that best practices in addiction treatment should include pharmacotherapy plus CBT or another evidence-based therapy, rather than usual clinical management or nonspecific counseling services.

## Key Points

**Question:**

Is cognitive behavioral therapy associated with improved outcomes for alcohol and other substance use disorders in the context of pharmacotherapy for addiction?

**Findings:**

This systemic review and meta-analysis including 30 studies found that combined cognitive behavioral therapy and pharmacotherapy was associated with increased benefit compared with usual care and pharmacotherapy. Cognitive behavioral therapy did not perform better than another evidence-based modality in this context or as an add-on to combined usual care and pharmacotherapy.

**Meaning:**

These findings suggest that best practices in addiction treatment should include pharmacotherapy plus cognitive behavioral therapy or another evidence-based therapy, rather than usual clinical management or nonspecific counseling services.

## Teaser

This meta-analysis and systemic review assess combined cognitive behavioral therapy and pharmacotherapy for alcohol or other substance use disorders in adults.

## Introduction

Substance use disorders (SUDs) represent a pressing public health concern, which calls for clinicians and scientists to identify and implement best practices in treatment.^1,2^ To that end, the combination of pharmacological and behavioral interventions has long been considered the criterion standard in addiction care,^3,4,5^ although differences between best practices for alcohol use disorder (AUD) and SUD have been noted.^6^ For SUDs without a US Food and Drug Administration (FDA)–approved pharmacotherapy, such as cocaine, methamphetamine, and cannabis, behavioral treatments are the principal approach.^7^ Cognitive behavioral therapy (CBT) is a first-line behavioral approach for treating AUD and other SUDs (AUD/SUD).^8^ Cognitive behavioral therapy is a time-limited, multisession intervention that targets cognitive, affective, and environmental risks for substance use and provides training in behavioral self-control skills to help an individual achieve and maintain abstinence or harm reduction.

Despite the importance of combined pharmacological and behavioral interventions for AUD/SUD, few meta-analyses on this intervention approach have been performed. Typically, meta-analytic reviews in the AUD/SUD literature have been conducted on specific pharmacotherapies,^9^ groups of pharmacotherapies,^10,11,12^ or specific behavioral interventions, such as CBT. As a result, the evidence-informed guideline will relate only to the selection of a single, stand-alone therapy, whether pharmacological or behavioral, and not their combination. For example, in a review of 122 clinical trials of AUD pharmacotherapies delivered in outpatient settings,^10^ the authors could not conclude about the efficacy of pharmacotherapies when combined with a behavioral cointervention.

The meta-analytic evidence on CBT supports efficacy at short- and long-term follow-ups.^13^ In an early review (1999) of 26 studies by Irvin et al,^14^ the authors found CBT to be generally effective across a range of conditions, but effect sizes were roughly 5 times higher when CBT was combined with pharmacotherapy than when delivered as a stand-alone intervention. This subgroup analysis was based on 4 studies and should therefore be interpreted with caution.^14^ In 2009, Magill and Ray^15^ followed up this work with a meta-analysis of 53 CBT clinical trials, reporting a similar overall effect size and a larger effect when CBT was combined with pharmacotherapy than when delivered alone, but the difference in effect-size magnitude between groups was smaller than observed in the previous review including 13 studies.

The goal of this meta-analysis is to provide an up-to-date and comprehensive review of CBT in conjunction with pharmacotherapy for AUD/SUD. This meta-analysis provides effect-size estimates across 3 distinct subgroups that may be informative to best-practice guidelines or individual clinician decision-making: (1) CBT plus pharmacotherapy compared with usual care (eg, clinical management, nonspecific drug counseling) plus pharmacotherapy, (2) CBT plus pharmacotherapy compared with another specific therapy (eg, motivational enhancement therapy, contingency management) plus pharmacotherapy, and (3) CBT added to usual care and pharmacotherapy compared with usual care and pharmacotherapy alone. Sensitivity analyses included tests of heterogeneity, study influence, and publication bias. Given the robust literature on CBT for addiction,^15,16^ the critical role of pharmacotherapy in addiction treatment,^1,2,17,18,19^ and the notion that combined treatments may be most effective,^3,17,18^ this meta-analytic review seeks to inform clinical practice and best-practice guidelines for addiction.

## Methods

### Literature Search Strategy

A literature search was conducted by a trained research assistant (J.W.) through July 31, 2019, as part of a broader meta-analysis project on CBT for substance use. First, we performed an all-fields search by treatment (*cognitive behavioral therapy* OR *relapse prevention* OR *coping skills training*), outcome (*alcohol* OR *cocaine* OR *methamphetamine* OR *stimulant* OR *opiate* OR *heroin* OR *opioid* OR *marijuana* OR *cannabis* OR *illicit drug* OR *substances* OR *dual disorder* OR *polysubstance* OR *dual diagnosis*), and study terms (*efficacy* OR *randomized controlled trial* OR *randomized clinical trial*) in the PubMed database. Then, we searched the Cochrane Register, Embase, and EBSCO databases (ie, MEDLINE and PsychINFO). Abstract screening was performed by 2 raters in abstrkr.^20^ A bibliographic search of CBT reviews was also performed to identify any candidate studies not identified by the original search methods^14,16,21,22,23,24^ This study followed the Preferred Reporting Items for Systematic Reviews and Meta-analyses (PRISMA) reporting guideline.

The Figure summarizes study inclusion for the present report on combined CBT and pharmacological interventions for adult AUD/SUD (PRISMA diagram). The final meta-analytic sample consisted of 30 studies and 62 effect sizes. No studies that fit the search criteria have been published since 2016. The protocol for this meta-analysis was not registered but was scientifically reviewed at the National Institutes of Health.

**Figure.**
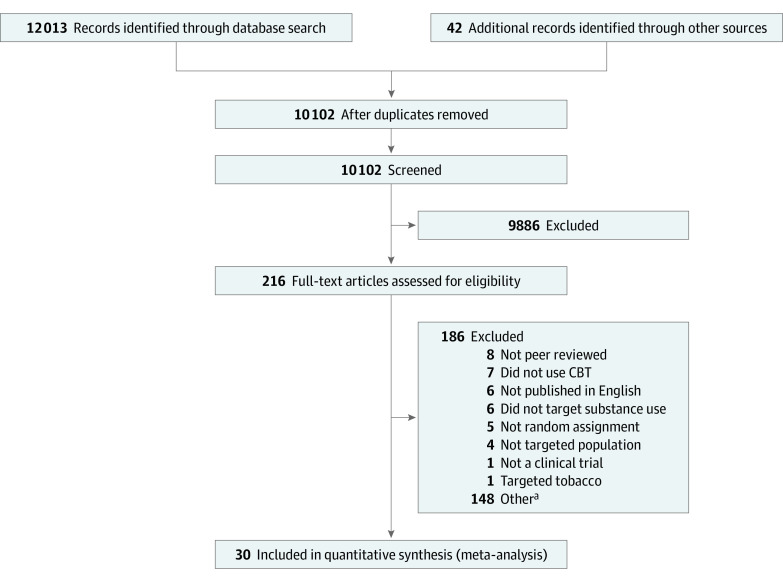
Flow of Study Inclusion (PRISMA Diagram) CBT indicates cognitive behavioral therapy. ^a^Includes studies diverted to other meta-analytic reports on CBT without pharmacotherapy, technology-delivered CBT, and CBT with dual-disorder populations.

### Primary Study Inclusion

Studies were English-language, peer-reviewed articles published from January 1, 1990, through July 31, 2019. Studies were primary outcome reports of randomized clinical trials. Given the importance of experimental contrast type in estimating effect-size magnitude in clinical trials,^25,26^ we used this design factor as a primary subgroup variable. Studies were included if they targeted adult populations (aged ≥18 years) meeting criteria for AUD or other drug use disorder (ie, *Diagnostic and Statistical Manual of Mental Disorders,* Third Edition Revised through Fifth Edition) or problematic use.^27^ Treatment must have been identified as either cognitive behavioral or relapse prevention. Concomitant treatment with pharmacotherapy for AUD/SUD was required for inclusion. Studies of CBT delivered in either individual or group format were included.

### Primary Study Characteristic Variables

Several study characteristic variables were examined in this meta-analysis. Study-level descriptors were mean (SD) age, percentage of female participants, percentage of white participants, percentage of black participants, percentage of Latinx participants, primary drug outcome (ie, alcohol, cocaine/stimulants, opioids, other), substance use severity (ie, dependence, abuse, or heavy use), treatment length (ie, number of planned sessions), treatment delivery (ie, individual or group format), study context (ie, community sample, specialty substance use or mental health clinic, medical setting, college setting, criminal justice setting, or other setting), publication country (ie, United States or other), and study-level risk of bias.^28^ Effect-size subgroup variables were (1) type of experimental contrast (ie, CBT plus pharmacotherapy compared with usual care plus pharmacotherapy, CBT plus pharmacotherapy compared with another specific therapy plus pharmacotherapy, CBT added to usual care and pharmacotherapy compared with usual care and pharmacotherapy alone), (2) type of substance use outcome type (ie, frequency and quantity), and (3) outcome point (posttreatment or follow-up). Data extraction guidelines were detailed in a study codebook available on request. Data were extracted in 2 independent passes conducted by trained raters (L.A.R., L.R.M., and J.W.). Final data entry where disagreement was observed required a consensus review by another author (M.M.).

### Primary Study Outcome Variables

The standardized mean difference was used to measure efficacy outcomes in this meta-analysis. The Hedges *g* statistic includes a correction for a slight upward bias in the estimated population effect.^29^ Before pooling, effect sizes were weighted by the inverse of the estimate variance to allow larger studies more influence on the overall effect size.^30^ Primary studies typically provided data on more than 1 outcome; therefore, data for effect-size estimation were selected based on a decisional hierarchy in the following order: (1) biological assay measures, (2) measures of frequency or quantity in the form of means (SDs), (3) sample proportions, and (4) other outcomes (eg, diagnostic measures). Most studies reported posttreatment outcomes only (18 [60%]), and if multiple months of follow-up data were reported, the latest time point was selected. Effect sizes were reverse scored as needed (eg, number of days with alcohol use) such that a positive effect size indicated a positive treatment outcome. Finally, when univariate outcome data were not reported, test statistics were transformed using available formulae.^31^ Effect size magnitude was interpreted using the follow benchmarks: 0.2 indicates small; 0.5, medium; and 0.8, large.^32^

### Statistical Analysis

Data were analyzed through September 30, 2019. Comprehensive Meta-analysis, version 3.0,^33^ was used for all analyses. Effect sizes for alcohol and other drug use were pooled using a random-effects model where there was an assumed distribution for the population effect size with systematic and random sources of variability.^34^ The significance of the *Q* test determined whether statistically significant between-study heterogeneity was present, and the *I^2^* statistic provided a percentage of heterogeneity estimate, regardless of statistical significance. When *I^2^* estimates exceeded 50%,^33^ primary drug outcome was tested as an effect-size moderator. We conducted other sensitivity analyses, including trimmed estimates with influential studies (ie, a study that, if removed, would change the substantive interpretation of the pooled effect size) removed and tests for publication bias.

## Results

### Sample-Level Descriptive Data

The sample included 30 unique AUD/SUD randomized clinical trials^3,7,35,36,37,38,39,40,41,42,43,44,45,46,47,48,49,50,51,52,53,54,55,56,57,58,59,60,61,62^ that examined CBT in combination with some form of pharmacotherapy. The study publication range was 1992 to 2016. The median sample size was 82 participants, with a range of 30^35^ to 917.^3^ The primary substance targeted in these clinical trials was alcohol (15 [50%]), followed by cocaine (7 [23%]) and opioids (6 [20%]). The sample mean (SD) participant age was 39 (6) years, with a mean (SD) of 28% (12%) female participants. Although reporting of race and ethnicity were inconsistent, the mean (SD) percentages were as follows: 66% (26%) white (21 of 30 studies), 35% (28%) black (15 of 30 studies), and 9% (7%) Latinx (15 of 30 studies). Diagnostically, study inclusion primarily (ie, 95%) targeted individuals with abuse or dependence as defined by *Diagnostic and Statistical Manual of Mental Disorders* (Fourth Edition) criteria.^63^ The CBT portion of these combined interventions was 73% individual and 26% group delivered, and 1 study^36^ used a mixture of individual and group sessions. The mean number of planned sessions was 16 (range, 4-48), and recruitment contexts were primarily specialty substance use or mental health clinics (20 [68%]), medical settings (5 [16%]), and community advertising (5 [16%]). The following pharmacotherapies were examined in this review: naltrexone hydrochloride and/or acamprosate sodium (26 of 62 effect sizes [42%]), methadone hydrochloride or combined buprenorphine hydrochloride and naltrexone (Suboxone) (11 of 62 [18%]), disulfiram (5 of 62 [8%]), and another pharmacotherapy or a mixture of pharmacotherapies (20 of 62 [32%]) (see Table 1 and Table 2 for details). Study-level risk-of-bias assessment showed 18 studies (60%) were low risk.^28^ When studies were designated as unclear or high risk, this was typically owing to (1) the presence of baseline differences between conditions, (2) no report of blinding of personnel and participants, and (3) no report of blinding of outcome assessment. Finally, most studies (21 [70%]) were published in the United States. Of studies conducted outside the United States, the following countries were represented: Germany, Sweden, the Netherlands, Switzerland, Finland, China, and Australia.

**Table 1.** CBT Combined With Pharmacotherapy Efficacy at Posttreatment by Type of Contrast Condition

Source	No. of participants^a^	Study treatment (No. of sessions)	Contrast treatment	Medication used	Substance used	Outcome^b^	Risk of bias^c^	*g* statistic (SE)^d^
**CBT plus pharmacotherapy vs usual care plus pharmacotherapy**								
Balldin et al,^43^ 2003	56	CBT (9)	Supportive therapy	Naltrexone	Alcohol	Days used	Low	0.46 (0.27)
Balldin et al,^43^ 2003	56	CBT (9)	Supportive therapy	Naltrexone	Alcohol	Heaving drinking days	Low	0.52 (0.27)
Carroll et al,^44^ 1994	54	Relapse prevention (12)	Clinical management	Desipramine hydrochloride	Cocaine	Days abstinent	Low	0.21 (0.27)
Carroll et al,^45^ 1998	53	CBT (12)	Clinical management	Disulfiram	Alcohol	Continuous weeks abstinent	Unclear	0.18 (0.28)
Carroll et al,^45^ 1998	53	CBT (12)	Clinical management	Disulfiram	Cocaine	Continuous weeks abstinent	Unclear	0.47 (0.28)
O’Malley et al,^46^ 1992	52	Relapse prevention (12)	Supportive therapy	Naltrexone	Alcohol	Days used	High	0.01 (0.29)
O’Malley et al,^46^ 1992	52	Relapse prevention (12)	Supportive therapy	Naltrexone	Alcohol	Drinks per drinking day of consumption	High	0.60 (0.44)
O’Malley et al,^47^ 2003	190	CBT (10)	Primary care management	Naltrexone	Alcohol	Days abstinent	Unclear	0.06 (0.15)
O’Malley et al,^47^ 2003	190	CBT (10)	Primary care management	Naltrexone	Alcohol	Drinks per drinking day	Unclear	0.04 (0.15)
Schmitz et al,^48^ 2001	44	Relapse prevention (20)	Drug counseling	Naltrexone	Cocaine	Percentage with negative urine screen	Unclear	2.20 (1.10)
Schmitz et al,^49^ 2004	40	Relapse prevention (20)	Drug counseling	Naltrexone	Alcohol	Days used	Unclear	0.49 (0.32)
Schmitz et al,^49^ 2004	40	Relapse prevention (20)	Drug counseling	Naltrexone	Cocaine	Percentage with positive urine screen	Unclear	−0.26 (0.31)
Schmitz et al,^49^ 2004	40	Relapse prevention (20)	Drug counseling	Naltrexone	Alcohol	Drinks per day of consumption	Unclear	0.68 (0.32)
Wetzel et al,^37^ 2004	121	CBT (24)	Group counseling	Nefazodone	Alcohol	Days abstinent	Low	0.13 (0.20)
Wetzel et al,^37^ 2004	121	CBT (24)	Group counseling	Nefazodone	Alcohol	Consumption, g/d	Low	0.17 (0.20)
**CBT plus pharmacotherapy vs specific therapy plus pharmacotherapy**								
Anton et al,^50^ 2005	80	CBT (12)	Motivational enhancement therapy	Naltrexone	Alcohol	Days abstinent	Low	0.57 (0.23)
Anton et al,^50^ 2005	80	CBT (12)	Motivational enhancement therapy	Naltrexone	Alcohol	Drinks per drinking day	Low	0.19 (0.22)
Carroll et al,^45^ 1998	51	CBT (12)	12-step facilitation	Disulfiram	Alcohol	Continuous weeks abstinent	Unclear	−0.06 (0.28)
Carroll et al,^45^ 1998	51	CBT (12)	12-step facilitation	Disulfiram	Cocaine	Continuous weeks abstinent	Unclear	0.18 (0.28)
Carroll et al,^7^ 2004	60	CBT (12)	Interpersonal psychotherapy	Disulfiram	Cocaine	Percentage with positive urine screen	Unclear	0.25 (0.26)
Davidson et al,^51^ 2007	149	Broad-spectrum treatment (12)	Motivational enhancement therapy	Naltrexone	Alcohol	Days abstinent	Unclear	0.25 (0.16)
Davidson et al,^51^ 2007	149	Broad-spectrum treatment (12)	Motivational enhancement therapy	Naltrexone	Alcohol	Heavy drinking days	Unclear	0.05 (0.16)
De Wildt et al,^52^ 2002	170	Brief CBT (7)	Motivational enhancement	Acamprosate	Alcohol	Days abstinent	Low	−0.09 (0.16)
Epstein et al,^41^ 2003	95	CBT (12)	Contingency management	Methadone	Cocaine	Times use per day	Low	−0.61 (0.21)
Ling et al,^53^ 2013	102	CBT (16)	Contingency management	Buprenorphine	Opioid	Percentage with negative urine screen	Low	−0.10 (0.20)
Oslin et al,^54^ 2008	81	CBT (18)	BRENDA^e^	Naltrexone	Alcohol	Heavy drinking days	High	0.76 (0.24)
Otto et al,^55^ 2014	78	CBT for interoceptive cues (15)	Cocaine collaborative individual drug counseling	Methadone	Opioid	Percentage with negative urine screen	Low	−0.13 (0.34)
Pettinati et al,^56^ 2008	82	CBT (12)	BRENDA^e^	Naltrexone	Poly drug use	Percentage with negative urine screen	Unclear	0.00 (0.22)
Rawson et al,^57^ 2002	60	Group CBT (48)	Contingency management	Methadone	Cocaine	Percentage with negative urine screen	Low	−0.44 (0.29)
**CBT plus usual care plus pharmacotherapy vs usual care plus pharmacotherapy**								
Anton et al,^3^ 2006^f^	917	Combined behavioral intervention (20) plus MM	MM	Acamprosate and/or naltrexone	Alcohol	Days abstinent	Low	−0.06 (0.11)
Anton et al,^3^ 2006^f^	917	Combined behavioral intervention (20) plus MM	MM	Acamprosate and/or naltrexone	Alcohol	Heavy drinking days	Low	0.08 (0.11)
Dürsteler-MacFarland et al,^35^ 2013	30	Group CBT (12) plus DAM	DAM	Methylphenidate plus diacetylmorphin	Cocaine	Percentage with negative urine screen	Low	−1.80 (0.42)
Epstein et al,^41^ 2003	97	CBT (12) plus MM	MM	Methadone	Cocaine	Times used per day	Low	−0.14 (0.20)
Ling et al,^53^ 2013	104	CBT (16) plus MM	MM	Buprenorphine	Opioid	Percentage with negative urine screen	Low	−0.03 (0.20)
Morgenstern et al,^40^ 2012	102	Modified behavioral self-control therapy (12) plus BBCET	BBCET	Naltrexone	Alcohol	Heavy drinking days	Low	2.90 (0.30)
Oslin et al,^54^ 2008	81	CBT (18) plus MM	MM	Naltrexone	Alcohol	Days drinking	High	0.67 (0.24)
Pan et al,^36^ 2015	240	CBT (32) plus MMT	MMT	Methadone	Opioid	Percentage with negative urine screen	Low	0.30 (0.13)
Rawson et al,^57^ 2002	60	Group CBT (48) plus MMT	MMT	Methadone	Cocaine	Percentage with negative urine screen	Low	0.44 (0.31)
Scherbaum et al,^42^ 2005	73	Group CBT (20) plus MMT	MMT	Methadone	Opioid	Percentage with positive urine screen	Low	−0.12 (0.23)
Schmitz et al,^58^ 2008	53	CBT (12) plus clinical management	Clinical management	Levodopa	Cocaine	Percentage with negative urine screen	Low	0.35 (0.36)
Tucker et al,^59^ 2004	97	Group relapse prevention (12) plus case management	Case management	Naltrexone	Opioid	Days used	Low	0.16 (0.20)

Abbreviations: BBCET, brief behavioral compliance enhancement treatment; CBT, cognitive behavioral therapy; DAM, diacetylmorphine maintenance; MM, medication management; MMT, methadone maintenance treatment; SE, standard error.

^a^ If an arm of the trial did not contribute an effect contrast, the study-level sample size was adjusted.

^b^ Negative outcomes such as days used or number of times used per day were reverse scored such that a positive effect estimate would reflect a positive treatment outcome.

^c^ Calculated using the Cochrane Risk of Bias Tool.^28^

^d^ Hedges *g* includes a correction for a slight upward bias in the estimated population effect.^29^ Before pooling, effect sizes were weighted by the inverse of the estimate variance to allow larger studies more influence on the overall effect size.^30^ Effect size magnitude was interpreted using the following benchmarks: 0.2 indicates small; 0.5, medium; and 0.8, large.^32^

^e^ Described by Starosta et al.^64^

^f^ Collapsed COMBINE Study medication conditions to test central contrast of interest to this report.

**Table 2.** CBT Combined With Pharmacotherapy Efficacy at Follow-up by Type of Contrast Condition

Source	No. of patients^a^	Study treatment (No. of sessions)	Contrast treatment	Medication used	Substance used	Follow-up, mo	Outcome^b^	Risk of bias^c^	*g* statistic (SE)^d^
**CBT plus pharmacotherapy vs usual care plus pharmacotherapy**									
Heinala et al,^38^ 2001	63	Cognitive coping skills (4)	Supportive therapy	Naltrexone	Alcohol	5	Use, g/wk	Low	2.00 (0.31)
Wetzel et al,^37^ 2004	121	CBT (24)	Group counseling	Nefazodone	Alcohol	9	Days abstinent	Low	0.24 (0.20)
Wetzel et al,^37^ 2004	121	CBT (24)	Group counseling	Nefazodone	Alcohol	9	Use, g/d	Low	0.50 (0.20)
**CBT plus pharmacotherapy vs specific therapy plus pharmacotherapy**									
Ling et al,^53^ 2013	102	CBT (16)	Contingency management	Buprenorphine	Opioid	5	Percentage with negative urine screen	Low	−0.06 (0.20)
Ling et al,^53^ 2013	102	CBT (16)	Contingency management	Buprenorphine	Opioid	8	Percentage with negative urine screen	Low	−0.06 (0.20)
Otto et al,^55^ 2014	78	CBT for interoceptive cues (15)	Individual drug counseling	Methadone	Opioid	2	Percentage with negative urine screen	Low	−0.09 (0.38)
Rawson et al,^57^ 2002	60	Group CBT (48)	Contingency management	Methadone	Cocaine	9	Percentage with negative urine screen	Low	0.16 (0.28)
Saunders et al,^60^ 2015	50	Integrated CBT (12)	Individual addiction counseling	Multiple	Polydrug use	6	Days used	Unclear	0.36 (0.28)
De Wildt et al,^52^ 2002	170	Brief CBT (7)	Motivational enhancement	Acamprosate	Alcohol	6	Percentage abstinent	Low	−0.09 (0.29)
Longabaugh et al,^39^ 2009	99	Broad spectrum therapy (24)	Motivational enhancement	Naltrexone	Alcohol	12	Days used	Low	−0.44 (0.28)
Longabaugh et al,^39^ 2009	99	Broad spectrum therapy (24)	Motivational enhancement	Naltrexone	Alcohol	12	Heavy drinking, d	Low	0.34 (0.32)
**CBT plus usual care plus medication vs usual care plus medication contrast**									
Anton et al,^3^ 2006^e^	917	Combined behavioral intervention (20) plus MM	MM	Acamprosate and/or naltrexone	Alcohol	12	Days abstinent	Low	−0.00 (0.11)
Anton et al,^3^ 2006^e^	917	Combined behavioral intervention (20) plus MM	MM	Acamprosate and/or naltrexone	Alcohol	12	Heavy drinking, d	Low	0.04 (0.11)
Berner et al,^61^ 2014	109	CBT (20) plus MM	MM	Acamprosate or naltrexone	Alcohol	18	Time to first lapse	Unclear	0.08 (0.21)
Fiellin et al,^62^ 2013	141	CBT (12) plus physician management	Physician management	Buprenorphine	Opioid	3	Days abstinent	Low	−0.00 (0.17)
Ling et al,^53^ 2013	104	CBT (16) plus MM	MM	Buprenorphine	Opioid	5	Percentage with negative urine screen	Low	0.00 (0.20)
Ling et al,^53^ 2013	102	CBT (16) plus MM	MM	Buprenorphine	Opioid	8	Percentage with negative urine screen	Low	0.00 (0.20)
Rawson et al,^57^ 2002	60	Group CBT (48) plus MMT	MMT	Methadone	Cocaine	9	Percentage with negative urine screen	Low	0.76 (0.30)
Saunders et al,^60^ 2015	56	Integrated CBT (12) plus standard care	Standard care	Multiple	Polydrug use	6	Days used	Unclear	0.69 (0.27)
Scherbaum et al,^42^ 2005	73	Group CBT (20) plus MMT	MMT	Methadone	Opioid	6	Percentage with positive urine screen	Low	0.24 (0.23)
Tucker et al,^59^ 2004	97	Relapse prevention (12) plus case management	Case management	Naltrexone	Opioid	3	Days used	Low	−0.13 (0.22)

Abbreviations: CBT, cognitive behavioral therapy; MM, medication management; MMT, methadone maintenance treatment; SE, standard error.

^a^ If an arm of the trial did not contribute an effect contrast, the study-level sample size was adjusted.

^b^ Negative outcomes such as days used or number of times used per day were reverse scored such that a positive effect estimate would reflect a positive treatment outcome.

^c^ Calculated using the Cochrane Risk of Bias Tool.^28^

^d^ Hedges *g* includes a correction for a slight upward bias in the estimated population effect.^29^ Before pooling, effect sizes were weighted by the inverse of the estimate variance to allow larger studies more influence on the overall effect size.^30^ Effect size magnitude was interpreted using the following benchmarks: 0.2 indicates small; 0.5, medium; and 0.8, large.^32^

^e^ Collapsed COMBINE Study medication conditions to test central contrast of interest to this report.

Tables 1 and 2 describe each study with respect to key design characteristics and effect sizes and are separated by posttreatment and follow-up outcomes, respectively. For outcomes of interest, biological assay/frequency measures and quantity measures are considered primary, and when available both are reported in Tables 1 and 2. Finally, the sample was distributed in roughly equal thirds with respect to the types of conditions to which combined CBT and pharmacotherapy was compared; narrative results and Tables 1 and 2 are organized by these clinically informative subgroups, and given the distinctiveness of these comparator conditions, no overall pooled effect size is reported. For pictorial plot information, see eFigures 1 through 10 in the Supplement.

### CBT Plus Pharmacotherapy Compared With Usual Care Plus Pharmacotherapy

When CBT plus pharmacotherapy was compared with usual care plus pharmacotherapy, the effect for CBT on posttreatment frequency outcomes was small, homogeneous, and statistically significant (*g = *0.18 [95% CI, 0.01-0.35]; *P* = .04; τ^2^* = *0.00, *Q* > 0.05, *I^2^* = 0%),^37,43,44,45,46,47,48,49^ but only 1 study^37^ provided follow-up effect-size data (*g = *0.24 [95% CI, −0.15 to 0.62]). For quantity outcomes,^37,43,46,47,49^ effects were small to moderate, homogenous, and significant (*g = *0.28 [95% CI, 0.03-0.54]; *P* = .03; τ^2^* = *0.03; *Q* > 0.05; *I^2^* = 31%), and only 2 studies^37,38^ provided follow-up effect-size data (*g = *0.50 [95% CI, 0.01-0.89] and *g = *2.00 [95% CI, 1.40-2.60], respectively). Among the studies in this subgroup, no evidence was found for influential studies or publication bias.

### CBT Plus Pharmacotherapy Compared With Another Specific Therapy Plus Pharmacotherapy

Studies that compared CBT with another specific therapy suggested no unique benefit of adding CBT to pharmacotherapy compared with other evidence-based modalities. For posttreatment frequency outcomes, effects were homogeneous and nonsignificant (*g = *0.05 [95% CI, −0.13 to 0.23]; *P* = .58; τ^2^* = *0.03; *Q* > 0.05; *I^2^* = 35%).^7,45,50,51,52,53,55,56,57^ A similar pattern of frequency results was found at follow-up (*g = *−0.02 [95% CI, −0.29 to 0.26]; *P* = .89; τ^2^* = *0.03; *Q* > 0.05; *I^2^* = 11%).^39,52,53,57,60^ Quantity posttreatment outcomes were also nonsignificant but statistically heterogeneous (*g = *0.09 [95% CI, −0.42 to 0.60]; *P* = .74; τ^2^* = *0.23; *Q* < 0.05; *I^2^* = 84%).^41,50,51,54^ At follow-up, only Longabaugh et al^39^ reported quantity outcomes (*g = *0.34 [95% CI, −0.29 to 0.96]). Sensitivity analyses showed no influential studies or evidence of publication bias.

### CBT as an Add-on to Usual Care and Pharmacotherapy Compared With Usual Care and Pharmacotherapy Alone

The final subgroup of studies examined CBT as an add-on to combined usual care and pharmacotherapy and suggested no clear added benefit of CBT in this context. Frequency results were heterogeneous and nonsignificant after treatment (*g = *0.06 [95% CI, −0.22 to 0.34]; *P* = .67; τ^2^* = *0.13; *Q* < 0.05; *I^2^* = 76%)^3,35,36,42,53,54,57,58,59^ and at follow-up (*g = *0.17 [95% CI, −0.05 to 0.38]; *P* = .13; τ^2^* = *0.04; *Q* > 0.05; *I^2^* = 51%).^3,42,53,57,59,60,62^ Only 3 studies (all AUD trials) provided quantity effect-size data, and these studies showed very different measures of effect (Anton et al^3^: *g* = 0.08 [95% CI, −0.14 to 0.31]; Epstein et al^65^: *g = −*0.14 [95% CI, −0.54 to 0.25]; Morgenstern et al^40^: *g = *2.90 [95% CI, 2.32-3.48]). At follow-up, the effect size for quantity outcomes in the COMBINE Study^3^ was *g = *0.04 (95% CI, −0.19 to 0.26) for collapsed pharmacotherapy, medication management, and combined behavioral intervention conditions vs pharmacotherapy and medication management only conditions. Sensitivity analyses showed no evidence of publication bias but showed 2 influential studies^35,40^ (eFigure 3 in the Supplement).

### Primary Drug Target as a Moderator of Between-Study Heterogeneity

In this review of CBT combined with pharmacotherapy for AUD/SUD frequency and quantity outcomes after treatment and at follow-up, most effect estimates showed little to no statistical heterogeneity. This outcome suggests that the variation between studies within a given pooled effect size was random rather than systematic.^33^ The exception was CBT combined with pharmacotherapy in contrast to another specific therapy combined with pharmacotherapy in relation to posttreatment quantity outcomes (*Q* < 0.05; *I^2^* = 84%)^41,50,51,54^ and CBT as an addition to combined usual care and pharmacotherapy in relation to posttreatment (*Q* < 0.05; *I^2^* = 76%)^3,35,36,42,53,54,57,58,59^ and follow-up (*Q* > 0.05; *I^2^* = 51%)^3,42,53,57,59,60,62^ frequency outcomes. Given that the sample of studies targeted different substances, each primary drug was examined as a subgroup moderator, and residual *I^2^* values were reported. Results are presented in Table 3. These analyses show that pooled effect-size direction and/or magnitude varied by primary drug outcome in this review. However, residual heterogeneity was present in 3 of the 8 subgroups, suggesting that this a priori moderator was an informative variable in this meta-analysis but did not explain all the systematic variance between studies. As a result, random-effects estimates are a particularly appropriate metric for this review.

**Table 3.** Subgroup Analyses of Heterogeneous Pooled Effect Sizes

Subgroup	*g* statistic (SE)	Source	*I^2^,* %
Compared with another specific therapy plus pharmacotherapy in relation to posttreatment quantity outcomes	0.09 (0.26)^a^	^4^	84
Alcohol studies	0.31 (0.27)	^3^	67
Cocaine or stimulant studies	−0.61 (0.21)^b^	^1^	NA
Added to usual care plus pharmacotherapy in relation to posttreatment frequency outcomes	0.06 (0.14)^a^	^9^	76
Alcohol studies	0.28 (0.36)	^2^	87
Opioid studies	0.14 (0.09)	^4^	14
Cocaine or stimulant studies	−0.32 (0.67)	^3^	90
Added to usual care plus pharmacotherapy in relation to follow-up frequency outcomes	0.17 (0.11)^a^	^7^	51
Alcohol studies	0.02 (0.10)	^2^	0
Opioid studies	0.02 (0.01)	^4^	0
Cocaine or stimulant studies	0.76 (0.30)^b^	^1^	NA

Abbreviations: NA, not applicable; SE, standard error.

^a^ Indicates the a priori pooled estimates.

^b^ *P* < .05.

## Discussion

To our knowledge, this is the first targeted meta-analysis of CBT in combination with pharmacotherapy for adults with AUD and other SUDs to summarize the data in a manner relevant to clinical practice guidelines. Furthermore, we provided pooled effect size estimates by consumption outcome type and outcome point. Most of these subgroup estimates showed acceptable homogeneity, which suggests that the selected variables were informative effect-size modifiers for the sample of clinical trials reviewed. In the present study, a small and statistically significant effect size was observed across outcome type and time for CBT combined with pharmacotherapy when compared with usual care combined with pharmacotherapy. For context in interpreting this effect, meta-analyses among this patient population generally show effect sizes that are in the small-to-moderate range,^41,66,67^ and this includes effect sizes for pharmacological interventions.^11,68,69^ This contrast suggests that prescribing clinicians should favor CBT over usual care to improve clinical outcomes for addiction, in the context of pharmacotherapy.

The second subgroup contrast was combined CBT and pharmacotherapy compared with another specific therapy combined with pharmacotherapy. Here, results suggested no unique benefit of adding CBT to pharmacotherapy compared with other evidence-based behavioral modalities. Such modalities may include contingency management, motivation enhancement therapy, 12-step facilitation, and interpersonal therapy, all of which have received some level of empirical support for addiction,^70,71^ including meta-analytic support at various follow-up periods.^23^ The lack of superiority of CBT over other evidence-based behavioral treatment for addiction is consistent with our recent findings,^13^ and this meta-analysis extends this result to combined pharmacotherapy and behavioral treatments. Although there may be evidence of some advantage for contingency management,^72^ removal of contingency management trials in this review did not change our substantive conclusions. This suggests that CBT is not superior to other evidence-based behavioral treatments for addiction, yet when combined with the aforementioned superiority to usual care, we suggest that clinicians favor an evidence-based behavioral therapy, CBT or otherwise, in conjunction with pharmacological treatments.

The third contrast in this meta-analysis assessed CBT as an add-on to usual care and pharmacotherapy compared with usual care and pharmacotherapy alone. In interpreting these findings, several explanations come to mind. First, there was substantial heterogeneity in the effect sizes obtained in these studies, suggesting unique study-specific factors could further explain outcome variability. This speculation is supported by the 2 influential studies observed in this subgroup.^35,40^ Moreover, moderator analyses by primary drug target showed variability in effect-size direction and magnitude with effects for cocaine and stimulant studies showing a range from moderate and negative^35,41^ to large and positive^57,58^ effects. This variability may be due, in part, to the lack of FDA-approved pharmacotherapy for cocaine/stimulant use disorder.^73^ In other words, FDA approval in this case was potentially confounded with the primary drug target. Second, some studies reported participants’ poor adherence to the CBT protocol, which may directly affect outcome.^42,65^ Third, the COMBINE Study is a large trial, which reported no benefit of the combined behavioral intervention over medication management. Close inspection of the medication management protocol for this study suggests it was a systematic, intensive, and rather robust intervention^74,75^ not readily comparable to standard clinical care. Together, these findings speak to the difficulty of measuring the benefit of an add-on component in complex clinical settings where multiple interventions are simultaneously administered.

### Limitations

This study has some limitations to consider. First, our primary goal was to derive valid, random-effects estimates characterized by effect modifiers. In other words, the goal was to avoid combining apples and oranges,^76^ which is a common criticism against meta-analysis. Although we consider our subgroup approach a strength, some effect size estimates were composed of a small number of primary studies, and this may result in underpowered analyses. Second, there may be some concern about fidelity and other sources of variability in what constituted this sample of CBT interventions, given that a range of manual implementations were reviewed. Unfortunately, fidelity to behavioral intervention protocol is often poorly reported in the clinical trial literature^77^ and could not be consistently measured for the present study. Similarly, this meta-analysis did not account for medication compliance or dose as a potential source of variability. The exclusion of 6 non-English articles is another potential limitation of this study. Finally, study results should be considered in the context of what constitutes an optimal outcome in clinical research with adult AUD/SUD. We selected consumption measures and favored biological assay variables in SUD trials, but equally important may be improvements in overall functioning,^78^ in which behavioral therapies, such as CBT, may be more likely to demonstrate effects.^18,19^ Therefore, the degree to which the outcomes presented in this review reflect an ideal end point remains an ongoing discussion for the field.

## Conclusions

Current clinical and research practices suggest that the rationale for combining behavioral therapy and pharmacotherapy is to provide support and skills while the patient is waiting for the medication effects to become apparent, to enhance treatment adherence, to improve treatment and study retention, and to address symptoms and problems that the medication will not address (eg, skills building).^18,19,74^ In an effort to inform best practices in addiction treatment, the following take-home points are drawn from the results of this meta-analysis. First, our results suggest that prescribing clinicians should favor CBT over usual clinical management to ensure optimal clinical outcomes for addiction, in the context of pharmacotherapy. This conclusion is based in our comparison of CBT plus pharmacotherapy vs usual care plus pharmacotherapy. Second, CBT is not superior to other evidence-based behavioral treatments for addiction, yet in the context of its superiority to usual care, our findings suggest that clinicians should favor an evidence-based behavioral therapy, CBT or otherwise, in conjunction with pharmacological treatment. Third, the add-on benefit of CBT compared with pharmacotherapy and usual care was not clearly supported and suggests that benefit of CBT as an adjunct requires further investigation.

## Notes

Supplement. **eFigure 1.** Plot of Effect Size by Standard Error **eFigure 2.** Plot of Effect Size by Standard Error **eFigure 3.** Plot of Effect Size by Standard Error **eFigure 4.** CBT Combined With Pharmacotherapy Compared With Usual Care Combined With Pharmacotherapy, Frequency Outcomes at Post Treatment **eFigure 5.** CBT Combined With Pharmacotherapy Compared With Usual Care Combined With Pharmacotherapy, Quantity Outcomes at Post Treatment **eFigure 6.** CBT Combined With Pharmacotherapy Compared With Another Specific Therapy Combined With Pharmacotherapy, Frequency Outcomes at Post Treatment **eFigure 7.** CBT Combined With Pharmacotherapy Compared With Another Specific Therapy Combined With Pharmacotherapy, Frequency Outcomes at Follow-up **eFigure 8.** CBT Combined With Pharmacotherapy Compared With Another Specific Therapy Combined With Pharmacotherapy, Quantity Outcomes at Post Treatment **eFigure 9.** CBT Combined With Usual Treatment and Pharmacotherapy Compared With Usual Treatment Combined With Pharmacotherapy, Frequency Outcomes at Post Treatment **eFigure 10.** CBT Combined With Usual Treatment and Pharmacotherapy Compared With Usual Treatment Combined With Pharmacotherapy, Frequency Outcomes at Follow-up **eReferences.**
