# Supplementary material for: Combined Pharmacotherapy and Cognitive Behavioral Therapy for Adults With Alcohol or Substance Use Disorders: A Systematic Review and Meta-analysis
Source: JAMA Netw Open. 2020 Jun 19;3(6):e208279. doi: 10.1001/jamanetworkopen.2020.8279 (PMC7305524; doi:10.1001/jamanetworkopen.2020.8279)
Supplement: Supplement. — eFigure 1. Plot of Effect Size by Standard Error eFigure 2. Plot of Effect Size by Standard Error eFigure 3. Plot of Effect Size by Standard Error eFigure 4. CBT Combined With Pharmacotherapy Compared With Usual Care Combined With Pharmacotherapy, Frequency Outcomes at Post Treatment eFigure 5. CBT Combined With Pharmacotherapy Compared With Usual Care Combined With Pharmacotherapy, Quantity Outcomes at Post Treatment eFigure 6. CBT Combined With Pharmacotherapy Compared With Another Specific Therapy Combined With Pharmacotherapy, Frequency Outcomes at Post Treatment eFigure 7. CBT Combined With Pharmacotherapy Compared With Another Specific Therapy Combined With Pharmacotherapy, Frequency Outcomes at Follow-up eFigure 8. CBT Combined With Pharmacotherapy Compared With Another Specific Therapy Combined With Pharmacotherapy, Quantity Outcomes at Post Treatment eFigure 9. CBT Combined With Usual Treatment and Pharmacotherapy Compared With Usual Treatment Combined With Pharmacotherapy, Frequency Outcomes at Post Treatment eFigure 10. CBT Combined With Usual Treatment and Pharmacotherapy Compared With Usual Treatment Combined With Pharmacotherapy, Frequency Outcomes at Follow-up eReferences. [file jamanetwopen-3-e208279-s001.pdf]

Supplementary Online Content

Ray LA, Meredith LR, Kiluk BD, Walthers J, Carroll KM, Magill M. Combined pharmacotherapy and cognitive behavioral therapy for adults with alcohol or substance use disorders: a systematic review and meta-analysis. *JAMA Netw Open*. 2020;3(6):e208279. doi:10.1001/jamanetworkopen.2020.8279

eFigure 1. Plot of Effect Size by Standard Error
eFigure 2. Plot of Effect Size by Standard Error
eFigure 3. Plot of Effect Size by Standard Error
eFigure 4. CBT Combined With Pharmacotherapy Compared With Usual Care Combined With Pharmacotherapy, Frequency Outcomes at Post Treatment
eFigure 5. CBT Combined With Pharmacotherapy Compared With Usual Care Combined With Pharmacotherapy, Quantity Outcomes at Post Treatment
eFigure 6. CBT Combined With Pharmacotherapy Compared With Another Specific Therapy Combined With Pharmacotherapy, Frequency Outcomes at Post Treatment
eFigure 7. CBT Combined With Pharmacotherapy Compared With Another Specific Therapy Combined With Pharmacotherapy, Frequency Outcomes at Follow-up
eFigure 8. CBT Combined With Pharmacotherapy Compared With Another Specific Therapy Combined With Pharmacotherapy, Quantity Outcomes at Post Treatment
eFigure 9. CBT Combined With Usual Treatment and Pharmacotherapy Compared With Usual Treatment Combined With Pharmacotherapy, Frequency Outcomes at Post Treatment
eFigure 10. CBT Combined With Usual Treatment and Pharmacotherapy Compared With Usual Treatment Combined With Pharmacotherapy, Frequency Outcomes at Follow-up
eReferences.

This supplementary material has been provided by the authors to give readers additional information about their work.

©2020 Ray LA et al. *JAMA Network Open*.

eFigure 1. Plot of Effect Size by Standard Error

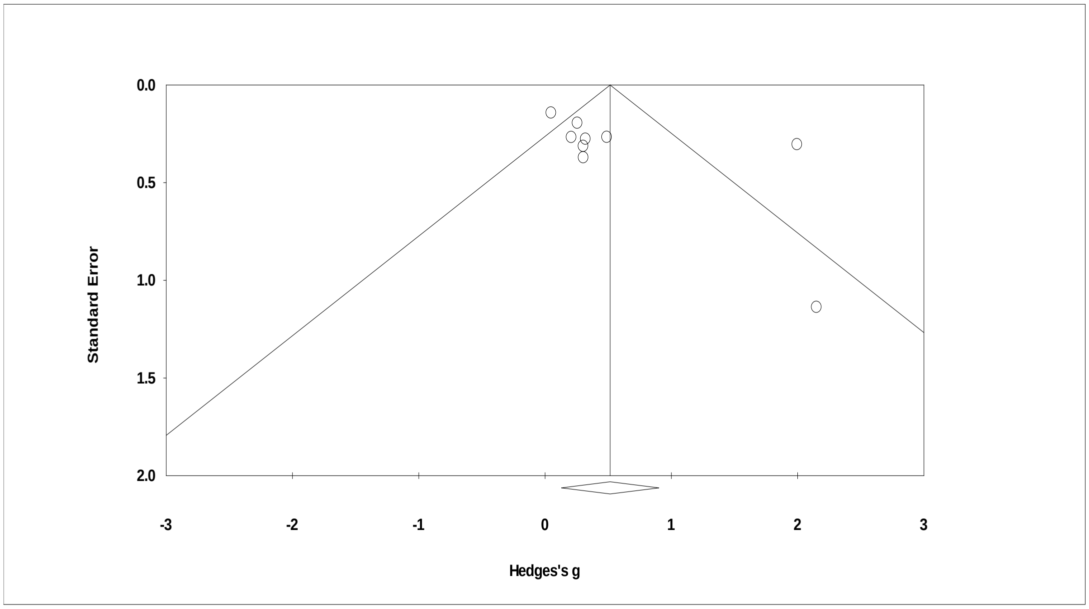

Assessment of bias in combined CBT and pharmacotherapy in contrast to combined usual care and pharmacotherapy. The plot shows some asymmetry, but the regression test was non-significant. Further, when two studies with outlier effects (Heinala et al.,[1] 2001; Schmitz et al.,[2] 2001) were removed, this did not change the substantive conclusions for this sub-group (g = 0.22: 95% CI = 0.04, 0.39, p = .014; Q > .05, I^2 = 0%; k = 7).

©2020 Ray LA et al. *JAMA Network Open*.

eFigure 2. Plot of Effect Size by Standard Error

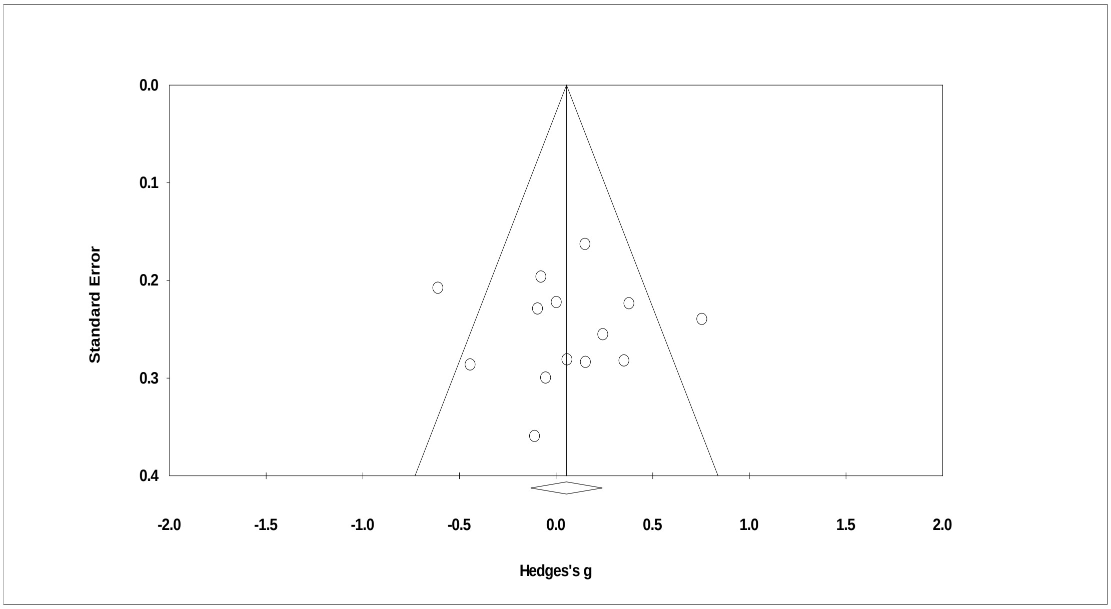

Assessment of bias in combined CBT and pharmacotherapy in contrast to another specific therapy combined with pharmacotherapy. This plot shows symmetry, and the regression test was nonsignificant.

©2020 Ray LA et al. *JAMA Network Open*.

eFigure 3. Plot of Effect Size by Standard Error

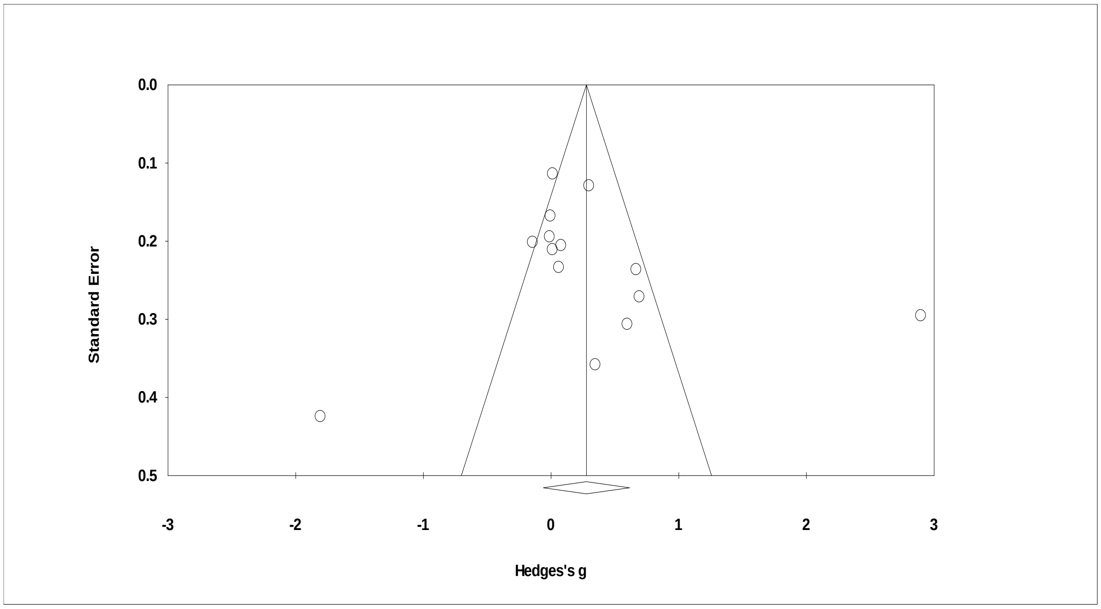

Assessment of bias in studies that added CBT to combined usual care and pharmacotherapy in contrast to usual care combined with pharmacotherapy alone. The plot shows some asymmetry, but the regression test was non-significant. When two studies with outlier effects (Dursteler-McFarland et al.,[3] 2013; Morgenstern et al.,[4] 2012) were removed, this resulted in a small and significant effect for CBT in this context (g = 0.17: 95% CI = 0.02, 0.31, p = .025; Q > .05, I^2 = 40%; k = 12).

©2020 Ray LA et al. *JAMA Network Open*.

eFigure 4. CBT Combined With Pharmacotherapy Compared With Usual Care Combined With Pharmacotherapy, Frequency Outcomes at Post Treatment*

Study name	Comparison	Hedges' g	Standard error	Lower limit	Upper limit
		Statistics for each study			
Balldin (2003)	CBT + Med v TAU + Med	0.460	0.269	-0.066	0.987
Carroll (1994)	CBT + Med v TAU + Med	0.211	0.270	-0.318	0.740
Carroll (1998)	CBT + Med v TAU + Med	0.181	0.277	-0.362	0.723
O'Malley (1992)	CBT + Med v TAU + Med	0.010	0.290	-0.559	0.578
O'Malley (2003)	CBT + Med v TAU + Med	0.064	0.145	-0.219	0.347
Schmitz (2001)	CBT + Med v TAU + Med	2.153	1.139	-0.080	4.386
Schmitz (2004)	CBT + Med v TAU + Med	0.493	0.315	-0.124	1.110
Wetzel (2004)	CBT + Med v TAU + Med	0.126	0.196	-0.258	0.509
		**0.180**	**0.085**	**0.013**	**0.347**

Hedges' g and 95% CI

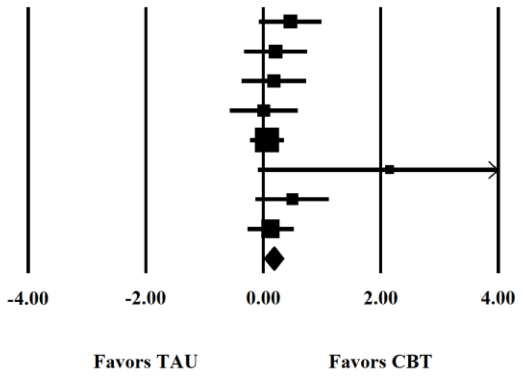

Notes. CBT = cognitive-behavioral therapy; TAU = treatment as usual; med = medication.
* No follow-up pooled estimate.

©2020 Ray LA et al. *JAMA Network Open*.

eFigure 5. CBT Combined With Pharmacotherapy Compared With Usual Care Combined With Pharmacotherapy, Quantity Outcomes at Post Treatment*

Study name	Comparison	Statistics for each study			
		Hedges' g	Standard error	Lower limit	Upper limit
Balldin (2003)	CBT + Med v TAU + Med	0.524	0.270	-0.004	1.053
O'Malley (1992)	CBT + Med v TAU + Med	0.603	0.441	-0.262	1.468
O'Malley (2003)	CBT + Med v TAU + Med	0.038	0.145	-0.245	0.321
Schmitz (2004)	CBT + Med v TAU + Med	0.683	0.319	0.057	1.308
Wetzel (2004)	CBT + Med v TAU + Med	0.172	0.196	-0.212	0.556
		**0.283**	**0.129**	**0.030**	**0.535**

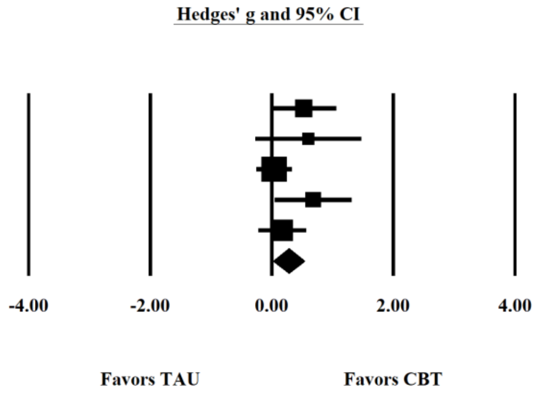

Notes. CBT = cognitive-behavioral therapy; TAU = treatment as usual; med = medication.
* No follow-up pooled estimate.

©2020 Ray LA et al. *JAMA Network Open*.

eFigure 6. CBT Combined With Pharmacotherapy Compared With Another Specific Therapy Combined With Pharmacotherapy, Frequency Outcomes at Post Treatment

Study name	Comparison	Statistics for each study			
		Hedges' g	Standard error	Lower limit	Upper limit
Anton (2005)	CBT + Med v specific + Med	0.573	0.226	0.129	1.016
Carroll (2004)	CBT + Med v specific + Med	0.245	0.256	-0.256	0.747
Carroll (1998)	CBT + Med v specific + Med	-0.064	0.281	-0.616	0.487
Davidson (2007)	CBT + Med v specific + Med	0.253	0.164	-0.068	0.574
De Wildt (2002)	CBT + Med v specific + Med	-0.091	0.156	-0.396	0.214
Ling (2013)	CBT + Med v specific + Med	-0.103	0.197	-0.489	0.283
Otto (2014)	CBT + Med v specific + Med	-0.125	0.343	-0.797	0.546
Pettinati (2008)	CBT + Med v specific + Med	0.004	0.223	-0.432	0.441
Rawson (2002)	CBT + Med v specific + Med	-0.441	0.287	-1.003	0.121
		**0.051**	**0.092**	**-0.130**	**0.232**

Hedges' g and 95% CI

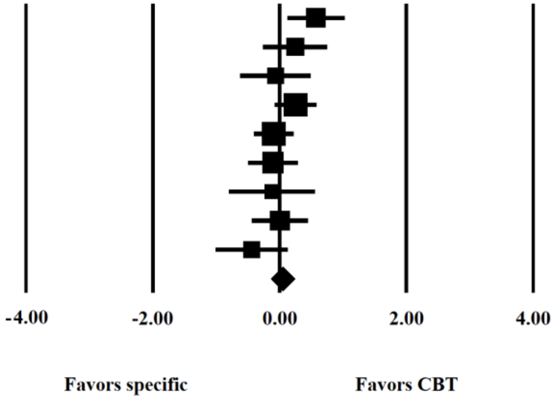

Notes. CBT = cognitive-behavioral therapy; med = medication

©2020 Ray LA et al. *JAMA Network Open*.

eFigure 7. CBT Combined With Pharmacotherapy Compared With Another Specific Therapy Combined With Pharmacotherapy, Frequency Outcomes at Follow-up

Study name	Comparison	Statistics for each study			
		Hedges' g	Standard error	Lower limit	Upper limit
De Wildt (2002)	CBT + Med v specific + Med	-0.094	0.285	-0.652	0.465
Longabuagh (2009)	CBT + Med v specific + Med	-0.437	0.279	-0.984	0.109
Otto (2014)	CBT + Med v specific + Med	-0.091	0.377	-0.829	0.647
Rawson (2002)	CBT + Med v specific + Med	0.155	0.284	-0.402	0.712
Saunders (2015)	CBT + Med v specific + Med	0.355	0.283	-0.199	0.909
		**-0.019**	**0.140**	**-0.293**	**0.256**

Hedges' g and 95% CI

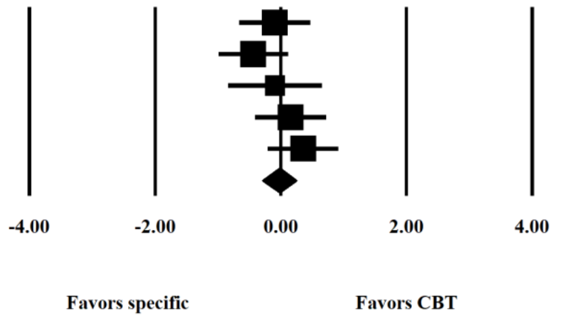

Notes. CBT = cognitive-behavioral therapy; med = medication

©2020 Ray LA et al. *JAMA Network Open*.

eFigure 8. CBT Combined With Pharmacotherapy Compared With Another Specific Therapy Combined With Pharmacotherapy, Quantity Outcomes at Post Treatment*

Study name	Comparison	Hedges' g	Standard error	Lower limit	Upper limit
Anton (2005)	CBT + Med v specific + Med	0.188	0.222	-0.247	0.623
Davidson (2007)	CBT + Med v specific + Med	0.052	0.163	-0.267	0.372
Epstein (2003)	CBT + Med v specific + Med	-0.608	0.208	-1.016	-0.200
Oslin (2008)	CBT + Med v specific + Med	0.757	0.240	0.287	1.228
		**0.088**	**0.259**	**-0.421**	**0.596**

Statistics for each study

Hedges' g and 95% CI

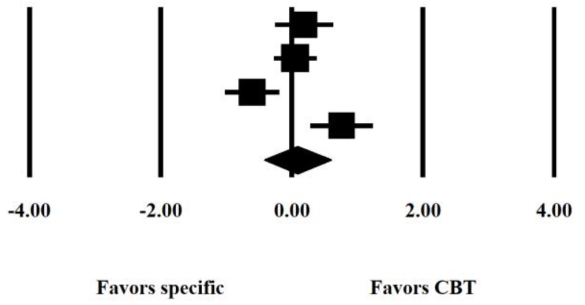

Notes. CBT = cognitive-behavioral therapy; med = medication
* No follow-up pooled estimate.

©2020 Ray LA et al. *JAMA Network Open*.

eFigure 9. CBT Combined With Usual Treatment and Pharmacotherapy Compared With Usual Treatment Combined With Pharmacotherapy, Frequency Outcomes at Post Treatment

Study name	Comparison	Statistics for each study			
		Hedges' g	Standard error	Lower limit	Upper limit
Anton (2006)	CBT + Med + TAU v Med + TAU	-0.056	0.114	-0.280	0.168
Dursteler-McFarland (2013)	CBT + Med + TAU v Med + TAU	-1.804	0.425	-2.636	-0.971
Ling (2013)	CBT + Med + TAU v Med + TAU	-0.027	0.195	-0.408	0.355
Oslin (2008)	CBT + Med + TAU v Med + TAU	0.669	0.237	0.205	1.133
Pan (2015)	CBT + Med + TAU v Med + TAU	0.300	0.129	0.046	0.554
Rawson (2002)	CBT + Med + TAU v Med + TAU	0.437	0.311	-0.173	1.047
Scherbaum (2005)	CBT + Med + TAU v Med + TAU	-0.117	0.234	-0.575	0.341
Schmitz (2008)	CBT + Med + TAU v Med + TAU	0.349	0.358	-0.353	1.052
Tucker (2004)	CBT + Med + TAU v Med + TAU	0.161	0.202	-0.236	0.558
		**0.061**	**0.143**	**-0.219**	**0.340**

Hedges' g and 95% CI

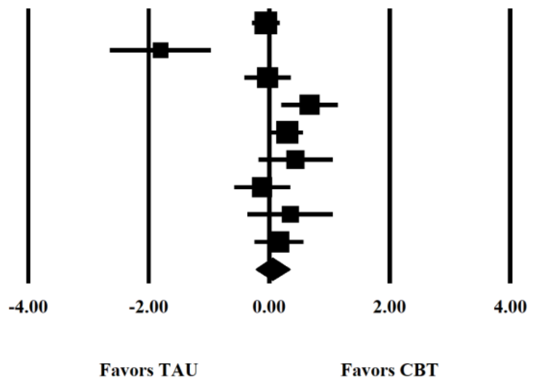

Notes. CBT = cognitive-behavioral therapy; TAU = treatment as usual; med = medication.

©2020 Ray LA et al. *JAMA Network Open*.

eFigure 10. CBT Combined With Usual Treatment and Pharmacotherapy Compared With Usual Treatment Combined With Pharmacotherapy, Frequency Outcomes at Follow-up*

Study name	Comparison	Statistics for each study				Hedges' g and 95% CI
		Hedges' g	Standard error	Lower limit	Upper limit	
Berner (2014)	CBT + Med + TAU v Med + TAU	0.081	0.206	-0.322	0.485	
Anton (2006)	CBT + Med + TAU v Med + TAU	-0.003	0.114	-0.227	0.221	
Fiellin (2013)	CBT + Med + TAU v Med + TAU	-0.002	0.168	-0.332	0.327	
Rawson (2002)	CBT + Med + TAU v Med + TAU	0.762	0.302	0.170	1.354	
Saunders (2015)	CBT + Med + TAU v Med + TAU	0.694	0.272	0.162	1.227	
Scherbaum (2005)	CBT + Med + TAU v Med + TAU	0.244	0.234	-0.215	0.703	
Tucker (2004)	CBT + Med + TAU v Med + TAU	-0.133	0.220	-0.563	0.297	
		**0.166**	**0.110**	**-0.049**	**0.380**	

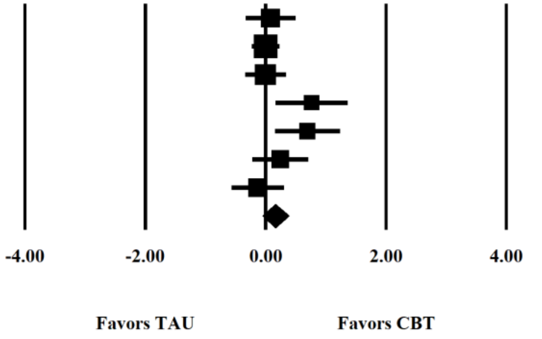

Notes. CBT = cognitive-behavioral therapy; TAU = treatment as usual; med = medication.
* No quantity post-treatment or follow-up pooled estimate.

©2020 Ray LA et al. *JAMA Network Open*.

eReferences.

1. Heinälä P, Alho H, Kiianmaa K, Lönnqvist J, Kuoppasalmi K, Sinclair JD. Targeted use of naltrexone without prior detoxification in the treatment of alcohol dependence: a factorial double-blind, placebo-controlled trial. J Clin Psychopharmacol. 2001;21(3):287-292. Medline:11386491 doi:10.1097/00004714-200106000-00006
2. Schmitz JM, Stotts AL, Rhoades HM, Grabowski J. Naltrexone and relapse prevention treatment for cocaine-dependent patients. Addict Behav. 2001;26(2):167-180.
3. Dürsteler-MacFarland KM, Farronato NS, Strasser J, et al. A randomized, controlled, pilot trial of methylphenidate and cognitive-behavioral group therapy for cocaine dependence in heroin prescription. J Clin Psychopharmacol. 2013;33(1):104-108. Medline:23277248 doi:10.1097/JCP.0b013e31827bfff4
4. Morgenstern J, Kuerbis AN, Chen AC, Kahler CW, Bux DA Jr, Kranzler HR. A randomized clinical trial of naltrexone and behavioral therapy for problem drinking men who have sex with men. J Consult Clin Psychol. 2012;80(5):863-875. Medline:22612306 doi:10.1037/a0028615
5. Balldin J, Berglund M, Borg S, et al. A 6-month controlled naltrexone study: combined effect with cognitive behavioral therapy in outpatient treatment of alcohol dependence. Alcohol Clin Exp Res. 2003;27(7):1142-1149.
6. Carroll KM, Rounsaville BJ, Gordon LT, et al. Psychotherapy and pharmacotherapy for ambulatory cocaine abusers. Arch Gen Psychiatry. 1994;51(3):177-187.
7. Carroll KM, Nich C, Ball SA, McCance E, Rounsavile BJ. Treatment of cocaine and alcohol dependence with psychotherapy and disulfiram. Addiction. 1998;93(5):713-727.
8. O'Malley SS, Jaffe AJ, Chang G, Schottenfeld RS, Meyer RE, Rounsaville B. Naltrexone and coping skills therapy for alcohol dependence: a controlled study. Arch Gen Psychiatry. 1992;49(11):881-887.
9. O'Malley SS, Rounsaville BJ, Farren C, et al. Initial and maintenance naltrexone treatment for alcohol dependence using primary care vs specialty care: a nested sequence of 3 randomized trials. Arch Intern Med. 2003;163(14):1695-1704.
10. Schmitz JM, Stotts AL, Rhoades HM, Grabowski J. Naltrexone and relapse prevention treatment for cocaine-dependent patients. Addict Behav. 2001;26(2):167-180.
11. Schmitz JM, Stotts AL, Sayre SL, DeLaune KA, Grabowski J. Treatment of cocaine-alcohol dependence with naltrexone and relapse prevention therapy. Am J Addict. 2009;13(4):333-341.
12. Wetzel H, Szegedi A, Scheurich A, et al; NeVeR Study Group. Combination treatment with nefazodone and cognitive-behavioral therapy for relapse prevention in alcohol-dependent men: a randomized controlled study. J Clin Psychiatry. 2004;65(10):1406-1413. Medline:15491246 doi:10.4088/JCP.v65n1017
13. Anton RF, Moak DH, Latham P, et al. Naltrexone combined with either cognitive behavioral or motivational enhancement therapy for alcohol dependence. J Clin Psychopharmacol. 2005;25(4):349-357.
14. Carroll KM, Fenton LR, Ball SA, et al. Efficacy of disulfiram and cognitive behavior therapy in cocaine-dependent outpatients: a randomized placebo-controlled trial. Arch Gen Psychiatry. 2004;61(3):264-272. Medline:14993114 doi:10.1001/archpsyc.61.3.264
15. Davidson D, Gulliver SB, Longabaugh R, Wirtz PW, Swift R. Building better cognitive-behavioral therapy: is broad-spectrum treatment more effective than motivational-enhancement therapy for alcohol-dependent patients treated with naltrexone? J Stud Alcohol Drugs. 2007;68(2):238-247.

16. De Wildt WA, Schippers GM, Van Den Brink W, Potgieter AS, Deckers F, Bets D. Does psychosocial treatment enhance the efficacy of acamprosate in patients with alcohol problems? Alcohol Alcohol. 2002;37(4):375-382.
17. Ling W, Hillhouse M, Ang A, Jenkins J, Fahey J. Comparison of behavioral treatment conditions in buprenorphine maintenance. Addiction. 2013;108(10):1788-1798.
18. Otto MW, Hearon BA, McHugh RK, et al. A randomized, controlled trial of the efficacy of an interoceptive exposure-based CBT for treatment-refractory outpatients with opioid dependence. J Psychoactive Drugs. 2014;46(5):402-411.
19. Pettinati HM, Kampman KM, Lynch KG, et al. Gender differences with high-dose naltrexone in patients with co-occurring cocaine and alcohol dependence. J Subst Abuse Treat. 2008;34(4):378-390.
20. Rawson RA, Huber A, McCann M, et al. A comparison of contingency management and cognitive-behavioral approaches during methadone maintenance treatment for cocaine dependence. Arch Gen Psychiatry. 2002;59(9):817-824.
21. Longabaugh R, Wirtz PW, Gulliver SB, Davidson D. Extended naltrexone and broad spectrum treatment or motivational enhancement therapy. Psychopharmacology (Berl). 2009;206(3):367-376. Medline:19639303 doi:10.1007/s00213-009-1615-3
22. Saunders EC, McGovern MP, Lambert-Harris C, Meier A, McLeman B, Xie H. The impact of addiction medications on treatment outcomes for persons with co-occurring PTSD and opioid use disorders. Am J Addict. 2015;24(8):722-731.
23. Epstein DH, Hawkins WE, Covi L, Umbricht A, Preston KL. Cognitive-behavioral therapy plus contingency management for cocaine use: findings during treatment and across 12-month follow-up. Psychol Addict Behav. 2003;17(1):73-82. Medline:12665084 doi:10.1037/0893-164X.17.1.73
24. Oslin DW, Lynch KG, Pettinati HM, et al. A placebo-controlled randomized clinical trial of naltrexone in the context of different levels of psychosocial intervention. Alcohol Clin Exp Res. 2008;32(7):1299-1308.
25. Anton RF, O'Malley SS, Ciraulo DA, et al; COMBINE Study Research Group. Combined pharmacotherapies and behavioral interventions for alcohol dependence: the COMBINE study: a randomized controlled trial. JAMA. 2006;295(17):2003-2017. Medline:16670409 doi:10.1001/jama.295.17.2003
26. Pan S, Jiang H, Du J, et al. Efficacy of cognitive behavioral therapy on opiate use and retention in methadone maintenance treatment in China: a randomised trial. PLoS One. 2015;10(6):e0127598. Medline:26107818 doi:10.1371/journal.pone.0127598
27. Scherbaum N, Kluwig J, Specka M, et al. Group psychotherapy for opiate addicts in methadone maintenance treatment: a controlled trial. Eur Addict Res. 2005;11(4):163-171. Medline:16110222 doi:10.1159/000086397
28. Schmitz JM, Mooney ME, Moeller FG, Stotts AL, Green C, Grabowski J. Levodopa pharmacotherapy for cocaine dependence: choosing the optimal behavioral therapy platform. Drug Alcohol Depend. 2008;94(1-3):142-150.
29. Tucker T, Ritter A, Maher C, Jackson H. A randomized control trial of group counseling in a naltrexone treatment program. J Subst Abuse Treat. 2004;27(4):277-288.
30. Berner MM, Wahl S, Brueck R, et al. The place of additional individual psychotherapy in the treatment of alcoholism: a randomized controlled study in nonresponders to anticraving medication-results of the PREDICT study. Alcohol Clin Exp Res. 2014;38(4):1118-1125.
31. Fiellin DA, Barry DT, Sullivan LE, et al. A randomized trial of cognitive behavioral therapy in primary care-based buprenorphine. Am J Med. 2013;126(1):74.e11-74.e17.

©2020 Ray LA et al. *JAMA Network Open*.
